# Behavioral Health Care Delivery Through Street Medicine Programs in California

**DOI:** 10.1007/s10597-023-01169-z

**Published:** 2023-08-01

**Authors:** Kimberly Y. Su, Brett J. Feldman, Corinne T. Feldman, Sonali Saluja, Alexis M. Coulourides Kogan, Michael R. Cousineau

**Affiliations:** Keck School of Medicine of USC, Los Angeles, CA USA

**Keywords:** Street medicine, Mental health, Substance use disorders, Homeless health, Behavioral health, Unsheltered homeless

## Abstract

Mental health and substance use disorders are prevalent among people experiencing homelessness. Street Medicine can reach unhoused people who face barriers to accessing healthcare in more traditional medical settings including shelter-based clinics. However, there is little guidance on best practices for mental health and substance use treatment through Street Medicine. The aim of the study was to describe behavioral health care through Street Medicine by analyzing data from the California Street Medicine Landscape survey and follow-up qualitative interviews. Most street medicine programs utilize non-psychiatrists to diagnose and treat mental health and substance use disorders, though the capacity to provide the level of care needed varies. There is a lack of street-based psychiatric clinicians and programs have difficulty making referrals to mental health and addiction services. This report shows that Street Medicine could serve as a strategy to expand access to behavioral health care for the unhoused.

## Introduction

Homelessness is among the nation’s most intractable social and public health problems. An estimated 582,500 people experienced homelessness in the United States in 2022, with 30% living in California (The U.S. Department of Housing & Urban Development, 2022). People experiencing homelessness (PEH) are more likely to have a broad range of health problems including serious mental illness (Fazel et al., 2008; Hossain et al., 2020; Iwundu et al., 2020; Koegel et al., 1988), substance use disorders (SUD) (Doran et al., 2018; Spinelli et al., 2016), and comorbid acute or chronic physical problems (Montgomery et al., 2016; Roncarati, Tiemeier, et al., 2021; Stringfellow et al., 2015; Vickery et al., 2021). PEH also have disproportionately high rates of mortality (Fine et al., 2022; Roncarati et al., 2018). Despite having higher needs, homeless people face formidable barriers to needed health care including lack of health insurance, transportation, costs, low health literacy, stigmatization, and limited capacity of service availability (Lawrence, 2020; Martins, 2008). These problems are particularly acute for the unsheltered homeless, whose living conditions on the streets expose them to unique challenges and obstacles.

Targeted health care programs for PEH were developed in the 1980s in response to the growing problem of urban homelessness (National Academies of Sciences, Engineering, and Medicine et al., 2018; National Association of Community Health Centers, 2019). These early programs deployed health care providers in shelters, transitional housing programs, drop-in centers, on vans and trailers. Many had outreach programs to seek out unhoused individuals who may be in need of services. Together, these approaches evolved into “street medicine” (SM), defined as the practice of bringing healthcare to people experiencing unsheltered homelessness (Withers, 2011). SM has expanded since its inception in the 1990s and is recognized as an important strategy for overcoming barriers to more traditional community-based health care (Lynch et al., 2022; Stefanowicz et al., 2021). SM differs from clinic-based care in that clinicians leave the four-wall brick and mortar setting to bring health care directly to the unsheltered patient on the street, under bridges, or in encampments—wherever they reside (Feldman, Feldman, et al., 2023; Feldman, Kim, et al., 2021).

SM provides a broad range of primary and urgent care services such as conducting physical examinations, performing diagnostic services including lab tests and imaging, providing certain medical procedures, and prescribing and dispensing medications. They also support patients through counseling, case management, referrals for specialty and behavioral health, health education, and connecting patients to social and housing services (Feldman, Feldman, et al., 2023; Feldman, Kim, et al., 2021).

There has been an increasing number of SM programs in the U.S and across the world that have shown success in providing needed care to PEH (Lynch et al., 2022). SM advocates attribute this success to its focus on building trust with their unhoused patients (Feldman, Kim, et al., 2021; Frankeberger et al., 2022; Howe et al., 2009; Withers, 2011). SM does so by employing designated team members who may have shared lived experiences with those in the patient population and providing nonjudgmental, individualized care to meet the patient where they are. Thus, SM may be well situated to provide street-based behavioral health care to unhoused individuals, many of whom have had experiences with the behavioral health system that have left them particularly distrustful of health care providers (Bhui et al., 2006). Currently, we know little about whether and how SM programs are approaching the management of behavioral health problems (Lo et al., 2021; McQuistion et al., 2003; Tsai et al., 2017). In this paper, the term “behavioral health” refers to mental health, psychiatric and SUDs, and any other diagnosis in the DSM-5. The objective of this study was to understand how and to what extent SM programs in California are providing behavioral health care. We report on the number of SM teams in California that provide behavioral health care, the types of behavioral health services offered, staffing patterns, and challenges in mobilizing behavioral health care for PEH. We also discuss implications for the expansion of behavioral health services more broadly to the unhoused population. This study was approved by the Institutional Review Board of the University of Southern California.

## Methods

**Fig. 1:**
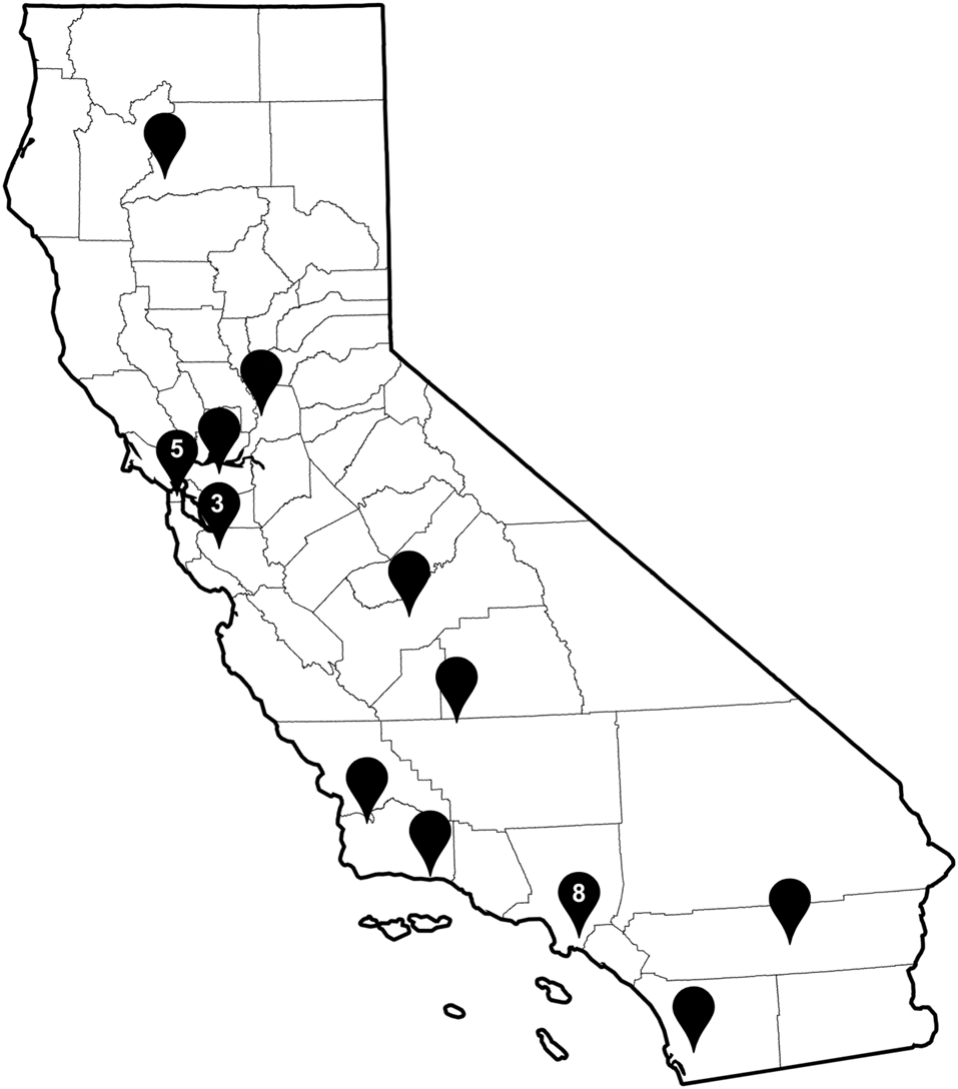
Locations of Street Medicine Programs that participated in The California Street Medicine Landscape Survey. Data collected from The California Street Medicine Landscape Survey conducted March and April 2022 (Feldman et al., 2023). Data visualization performed by Kimberly Su

Data for this report are derived from The California Street Medicine Landscape Report (Feldman et al., 2023) conducted between March and June of 2022. This was a sequential explanatory mixed-methods study consisting of a cross-sectional survey and qualitative stakeholder interviews with a selected group of SM teams in California. For the survey, a list of 38 organizations in California were identified as operating SM or street-based services and were invited to participate in the study. Each was sent a link to an electronic 41-item questionnaire which asked detailed information about program characteristics around staffing, funding, patient profiles, services offered, finances, and organizational affiliation. At the time of study invitation, nine programs reported that they did not provide SM and were excluded from the study. Of the remaining 29, three did not respond to several requests; 26 completed the survey, resulting in a response rate of 92%. However, only data from 25 SM programs were analyzed due to the newness of one program which did not have annualized figures to report. (See Fig. 1 for a graphic of the geographic locations of the SM programs that participated in the survey [Feldman et al., 2023]).

### Interviews

To supplement the survey analysis, we conducted interviews with program directors (n = 2), medical directors of healthcare services (n = 2), SM practitioners (n = 2) and a social worker (n = 1) from three distinct SM entities operating throughout LA County to gain qualitative insight into behavioral health treatment in SM not captured by the Landscape Survey. Interviews were conducted through video call except for one which was conducted as an in-person site visit. In the interviews, we asked the three following open-ended questions: (1) “How do you provide mental health, SUD, and psychiatric services on the street?” (2) “How are your SM programs staffed to accommodate provision of behavioral health services?” (3) “What is the referral process for behavioral health services?” Interviewees were asked to elaborate on successes, challenges, and specific examples for each of the three questions. In this paper, their responses are organized under the corresponding headings.

### Analysis

In this paper we analyze data from the Landscape Analysis pertaining to behavioral health using IBM SPSS Statistics. This includes the number of patients served who have behavioral health problems, types of behavioral health services provided, and behavioral health staffing patterns deployed in the teams. The interview data were analyzed using detailed notes followed by content analysis of the notes for each interview completed.

## Results

### Prevalence of Mental Disorders Among Street Medicine Patients

**Fig. 2:**
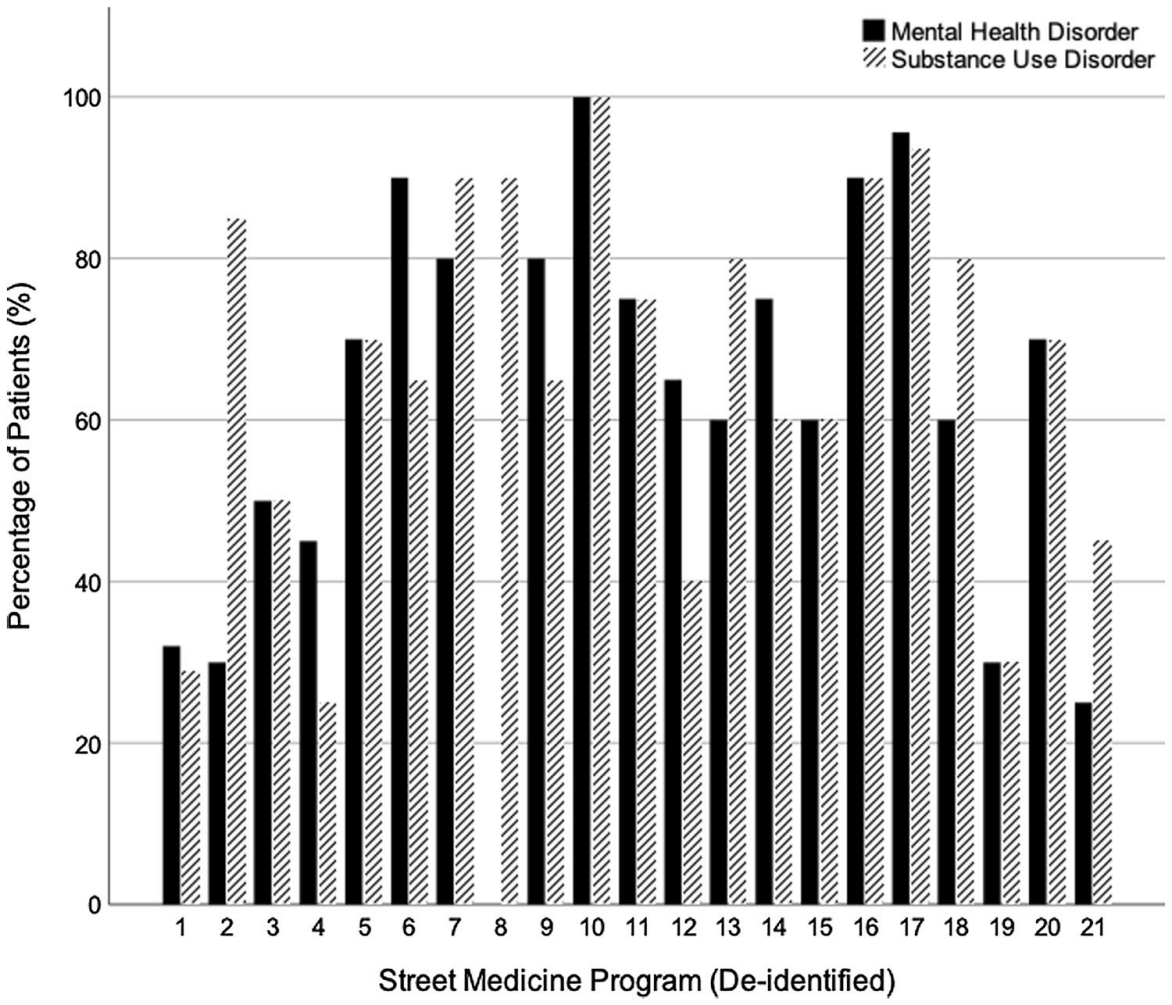
Percentage of Street-Based Patients with Mental Health and Substance Use Disorders. Not all SM programs responded to this question (n = 21). Four programs did not provide estimates for mental health disorder or SUD and one program only provided an estimate for SUD. Data collected from The California Street Medicine Landscape Survey conducted March and April 2022 (Feldman et al., 2023). Graph created using SPSS Statistics

Collectively, the 25 responding programs served 9,682 unduplicated patients in the 2021 fiscal year, ranging from 30 to 2,195 patients. Nearly three-quarters of the teams reported that at least half of their patients had any type of mental health disorder (n = 15); three-quarters similarly responded that more than half of their patient population had SUDs (n = 16). Qualitative interviews confirmed that many SM patients have co-occurring physical, mental health, and substance use conditions. (See Fig. 2 for percentage of street-based patients with mental health or SUDs).

### Mental Health Services

**Fig. 3:**
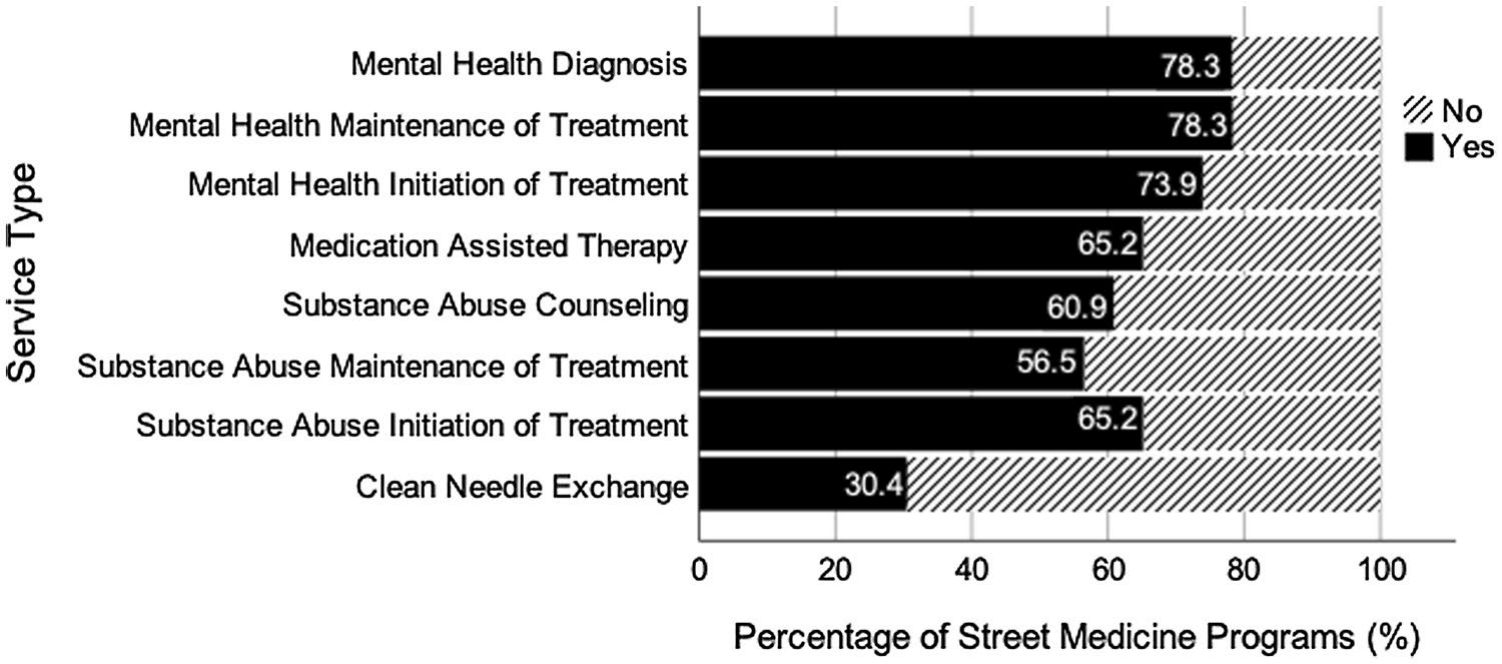
Provision of Various Mental Health Services in California Street Medicine Programs. Not all SM programs responded to this survey item (n = 23). Data collected from The California Street Medicine Landscape Survey conducted March and April 2022 (Feldman et al., 2023). Graph created using SPSS Statistics

The majority of SM programs provide some type of behavioral health services. Over three quarters (n = 18) report diagnosing mental health disorders; approximately three quarters report initiating (n = 17) or continuing (n = 18) treatment for a mental health condition. For SUD treatment, two-thirds initiate treatment (n = 15), two-thirds provide medication assisted therapy (MAT) (n = 15), with fewer but still more than half providing counseling (n = 14), initiating (n = 15) or maintenance (n = 13) treatment for SUD; less than a third provide clean needle exchange (n = 7). Only one SM program reported not providing mental health or SUD treatment of any type. (See Fig. 3 for Provision of various mental health services by California Street Medicine Programs).

In the interviews, one SM physician and program director used the term “bridge psychiatry” whereby primary care providers (MD/DO/PA/NP) provide street-based mental health treatment to stabilize patients before referral and transfer to a psychiatric specialist when possible. This involves utilizing buprenorphine or suboxone for opioid dependence, or antipsychotic medications including long-acting injectable (LAI) antipsychotics which are discussed in the following paragraph. The interviewee emphasized the need for bridge psychiatry, due to the lack of psychiatric specialists in SM and to help patients begin treatment who would otherwise experience treatment delays due to the referral process, which may take days to weeks, if at all available.

Interview participants from all three interviewed SM programs reported using LAI antipsychotic medications, which are administered intramuscularly and have the advantage of delivering a continuous dose of antipsychotic medication for a period of up to several weeks as opposed to oral antipsychotic medications which patients must take at least once per day. One interviewee described how many PEH have difficulty adhering to a daily oral medication regimen because pills are often lost or stolen, or may degrade while kept in tents, pockets or backpacks making injectables an attractive option for treating schizophrenia and some cases of bipolar disorder in PEH.

### Mental Health Staffing

Programs varied in how they staffed SM teams. There was no clear distinction between team members who were managing physical versus mental health complaints based on collected survey data which demonstrates an integrative approach to managing behavioral health in the context of primary care. Follow up interviews reported that SM team members involved in providing mental health care include community health workers, case managers, licensed clinical social workers, SUD counselors, medical assistants, nurses (RN, LVN), psychiatric nurse practitioners, physician assistants, primary care physicians, and psychiatrists.

**Fig. 4:**
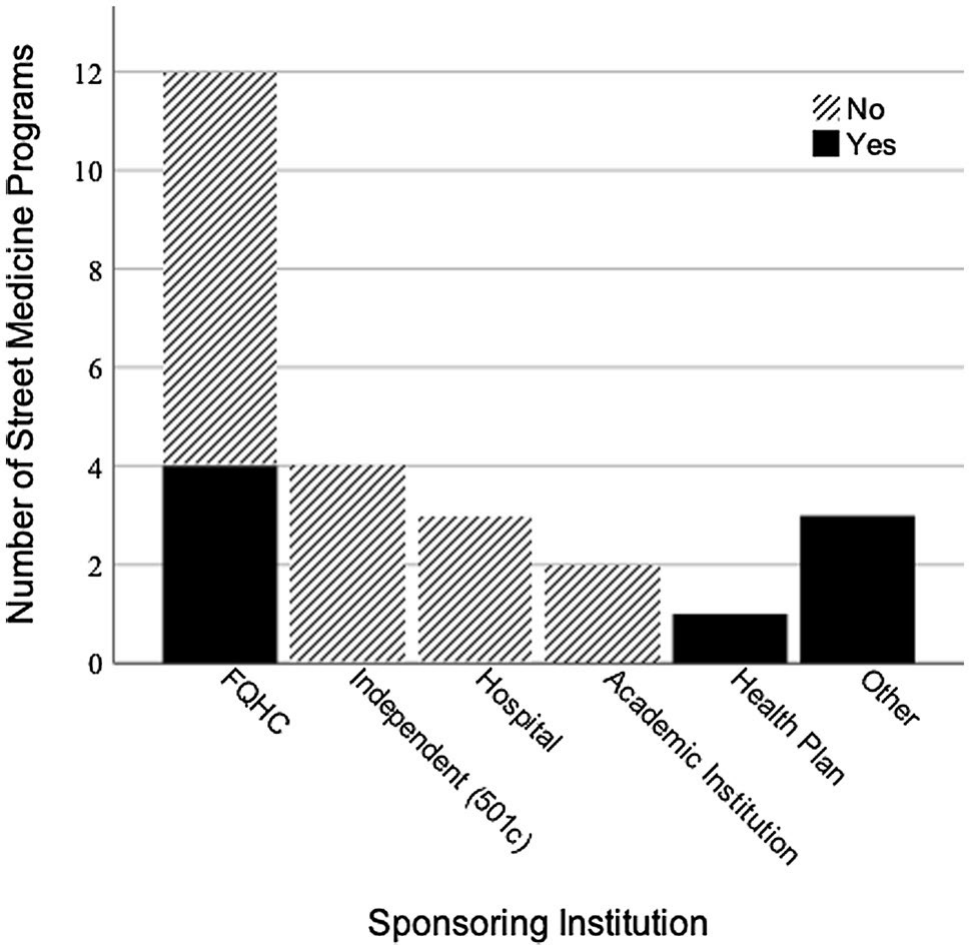
Street Medicine Staffing of Paid Psychiatrists by Sponsoring Institution. Other = County, City and County Department of Health, VA Healthcare System. Sources: Data collected from The California Street Medicine Landscape Survey conducted March and April 2022 (Feldman, et al., 2023). Graph created using SPSS Statistics

**Fig. 5:**
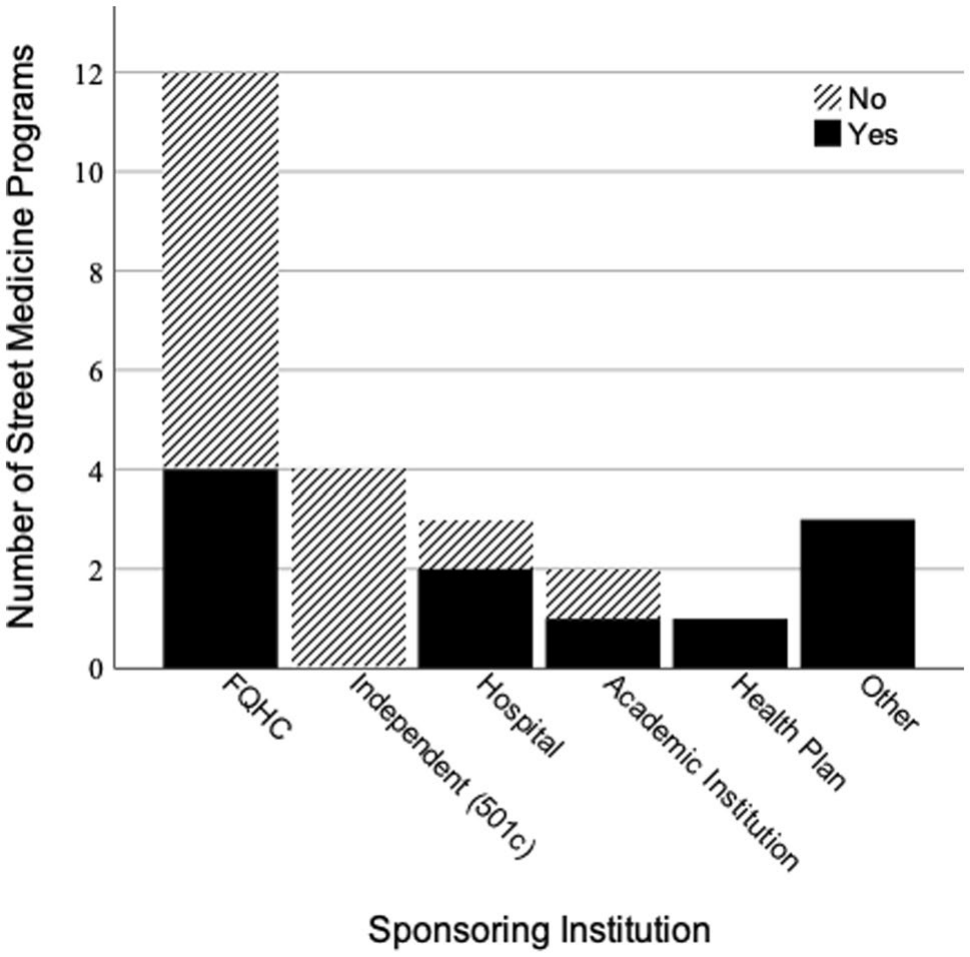
Street Medicine Staffing of Paid Social Workers by Sponsoring Institution. Other = County, City and County Department of Health, VA Healthcare System. Data collected from The California Street Medicine Landscape Survey conducted March and April 2022 (Feldman et al., 2023). Graph created using SPSS Statistics

Out of the 25 programs surveyed, eight employed a psychiatrist. Two programs had a full-time psychiatrist while the remaining six had part-time psychiatrists. The programs with part-time psychiatrists had Full Time Equivalents (FTE) of 40 h per week ranging 0.1–0.25 FTEs. This means that the six programs with a psychiatrist-staffed part-time had access to the psychiatrist ranging from 4 to 10 h per week. Four programs with a paid psychiatrist were sponsored by a federally qualified health center (FQHC), two by County Departments of Health, one by a health plan, and one by the Veterans Affairs (See Fig. 4 for SM staffing of paid psychiatrists by sponsoring institutions). Almost half of the programs (11 out of 25) had paid social workers (ASW or MSW, MFT, LCSW) many of whom are licensed to provide mental health counseling or support services. However, the California Street Medicine Landscape Survey did not ask if mental health counseling was being administered by the social workers (See Fig. 5 for SM staffing of paid social workers by sponsoring institutions).

All interviewees noted the importance of a multidisciplinary team of social workers, case managers, and community health workers on the SM team, especially for patients with mental health disorders, SUDs, or both. Community health workers may have shared lived experiences with homelessness, mental health disorders, or SUDs—allowing them to build relationships with patients based on shared experience and empathy. One interview participant described that the roles of the community health workers and case managers include reminding street-based patients to take their medications, delivering medications, and tracking down patients for the SM providers to provide services. These services are particularly important for patients with mental health disorders and SUDs who oftentimes need additional support with adhering to treatments.

### Transition of Treatment and Referrals

In the Landscape Survey, respondents were asked to rate their success in referring patients to mental health or addiction treatment programs and clean needle exchange services on a five-point likert scale (1 = not successful, 5 = always successful). Programs reported difficulty in successfully referring patients to both mental health and addiction treatment programs (M = 2.8, SD = 0.9); three programs reporting that they do not refer for these services. Programs reported less difficulty in referring to clean needle exchange services (M = 3.5, SD = 1.2) with seven programs reporting that they do not refer patients for this service. Similarly, when asked whether programs felt they had sufficient resources to support their patients’ needs (1 = definitely no, 5 = definitely yes), programs overall reported having insufficient resources in mental health or addiction treatment programs (M = 2.7, SD = 0.8), but slightly more sufficient resources with clean needle exchange services (M = 3.0, SD = 1.3). In the open-ended comments field in the California Landscape Survey, respondents referenced unsheltered patients having difficulty keeping appointments, or lack of means of contact such as phone number and address, and transportation barriers as patient reasons for referral difficulties. They also noted systemic issues such as lack of resources or organizations to refer their patients to, agencies not accepting new clients, and delays in referrals due to available appointments being too far out.

During the follow up interviews, one SM director explained their program’s goal which is to utilize their partnerships with community based residential and outpatient mental health and SUD programs to facilitate a “warm hand-off” to connect their street-based patients. Another SM physician from an FQHC based team operates out of a medically equipped van. Their team regularly refers patients with mental health needs to their own FQHC. Importantly, internally referred patients are entered into the clinic’s electronic medical record (EMR) system so medical information is readily available when the patient arrives at the site for behavioral health appointment. This physician also spoke on the difficulty of referring their street-based patients to inpatient psychiatric facilities which can be “unwelcoming” to unhoused patients.

Similarly, a SM program director reported that their homeless patients initially present at the program’s affiliated hospital emergency department. From there, they are connected to the SM team who can then follow-up on the patients’ medical, mental health, and SUD treatment needs once the patients are discharged from the hospital. Another interviewee reported that their program aims to provide services for all patients with different levels of health-related needs. Their program takes into consideration a patient’s specific circumstances and severity of illness. Based on this assessment, they are able to tailor a set of services to meet those needs. Another SM provider indicated that patients who are more “high-functioning” may be able to be stabilized and transition to care in a clinic, whereas another patient with more complex needs may need to be referred to a higher level of care. Regardless of the model, follow up interviews consistently emphasized the importance of their SM team being a consistent presence in their community and building trust with PEH.

## Discussion

Given the high prevalence of mental health disorders and SUDs amongst PEH, new ways of expanding access to behavioral health care will be important in a community’s efforts to address homelessness. SM is a promising strategy for bringing mental health care and SUD treatment to people experiencing homelessness and our study aimed to examine how this is being done in California. Our study found that most SM programs are diagnosing and providing some level of treatment for mental health and SUD by leveraging the interdisciplinary teams to integrate mental health and primary care services. This strategy has been documented in other studies as being a practical way of meeting the complex needs of unsheltered patients (Feldman et al., 2023; Jego et al., 2018; Lo et al., 2021). Many SM teams have incorporated treatment for SUD, including MAT—a critical service given the high rates of SUD among PEH—into the context of other primary care services (Feldman et al., 2023). Thus, SM can serve an important role in addressing SUD among PEH. Still, our study highlights the need to further understand barriers to treating SUD among SM programs who seem otherwise well positioned to bring this service to patients most in need. Previous regulatory barriers such as restrictions on mobile prescribing of methadone and buprenorphine (two medications used to treat opioid use disorder and in MAT) and limitations to NPs and PAs prescribing these medications have recently been lifted (U.S. Department of Health & Human Services, 2023). Thus, some SM programs may be unaware of these new efforts to expand access to MAT or providers may be unwilling to prescribe MAT due to inexperience or lack of training.

SM may also provide an important way of expanding access to LAI antipsychotics as a way to treat schizophrenia and other serious mental health problems. In the past decade, LAI antipsychotics have gained recognition in being effective in curbing nonadherence and preventing hospitalization and relapse (Abdel-Baki et al., 2022; Correll et al., 2016; Tiihonen et al., 2017). Further studies are needed to understand the extent to which SM programs are utilizing LAI antipsychotics, and to identify factors that prevent its current use in practice of Correl et al. (2016) identified factors including provider training, negative attitudes about LAIs, and resource and cost issues for the low utilization of LAIs for patients with schizophrenia in the general population.

McQuistion et al. (2003) discussed the clinical, administrative, academic, and advocacy roles that psychiatry has for homeless people with psychiatric disorders. There is a shortage in the workforce of US psychiatrists to meet the mental health needs of the general public (Satiani et al., 2018), and even more so for communities of lower socioeconomic status, such as PEH (McLaughlin et al., 2021). Our findings highlighted this disparity given the high prevalence of mental health and SUDs in the SM patient population and only a quarter of our SM program respondents reporting staffing of psychiatrists to help with complex diagnosis and management of mental health disorders in PEH. Results from the Landscape Analysis and our interviews highlighted that non-psychiatric providers in SM are filling the gap to address the mental health needs of unsheltered patients. Most non-psychiatric providers have experience in treating mental disorders such as depression and anxiety but may have less so for severe mental health disorders, though our survey and interviews were not able to parse out these kinds of distinctions. Given the shortage of psychiatrists in SM, further studies to understand the training and comfort levels of SM providers in management of psychiatric patients can aid in developing training for non-psychiatric SM providers in common behavioral health problems affecting PEH, which has the potential to ultimately improve mental health outcomes for PEH.

SM programs can benefit from better coordination and stronger partnerships with psychiatric and SUD providers in community-based organizations. Our data shows that primary care providers in SM are attempting to refer their patients for mental health and substance use treatment services but are generally not finding success, likely due to the shortage of psychiatric specialists in the workforce. In comparison, SM programs report having better success in referring their patients to clean needle exchange services, likely due to the fact that clean needle exchanges are run by non-clinicians. Exploration into the feasibility and acceptability of psychiatric and addiction telemedicine services for people experiencing unsheltered homelessness is an important area of future exploration. Further, consideration of how to best use the limited resources of a street-based psychiatrist is unknown.

Further research can be conducted to identify best practices and identify further challenges that stand in the way of mental health treatment through SM. Of note, best practices for each SM program may differ depending on the needs of specific communities, program funding and available resources.

## Limitations

The multi-methods design, high response rate, completeness of survey data, and inclusion of programs across the state of California highlight study rigor and importance of findings. Still, there are some limitations to our study. First, we focused on a single state so findings may not be generalizable to SM programs outside of California. However, SM programs across the nation are becoming increasingly more homogeneous in many of their activities as teams adopt standardized clinical approaches and best practices, based on the work of the Street Medicine Institute and better communication across programs. Second, the SM programs interviewed were all based in Los Angeles County. Nevertheless, these interviews were not meant to be representative, but rather aid in the clarification of activities and provide examples of quantitative results of the California Street Medicine Landscape Survey. Third, some respondents may have provided estimates in lieu of actual figures which potentially limits the quality of the data. However, since these represented less than 10% of the respondents, the conclusions would not vary if these responses were excluded.

## Conclusions and Recommendations

Our findings suggest that SM is a promising approach for behavioral health delivery for unsheltered people. As one of our interviewees said: “the reality of the lives of people experiencing homelessness warrants a specialized approach to healthcare delivery—that includes mental health, behavioral health, and substance use treatment.” The ramifications of the US shortage of behavioral health professionals are amplified for vulnerable populations such as PEH. SM could be an effective way to expand access to behavioral health care to PEH, both directly on the street and through effective referrals to inpatient and outpatient care in community-based sites. Improving behavioral health care access and outcomes for PEH could help local cities better transition unsheltered individuals from the streets to permanent supportive housing. We recommend the development of innovative strategies for providing behavioral health through SM, but also that these strategies be rigorously studied and evaluated to allow for the development of evidence-based practices that can be tested, replicated, reimbursed, and institutionalized.
